# The effect of D-cycloserine on brain connectivity over a course of pulmonary rehabilitation – A randomised control trial with neuroimaging endpoints

**DOI:** 10.1371/journal.pone.0323213

**Published:** 2025-06-02

**Authors:** Sarah L. Finnegan, Olivia K. Harrison, Martyn Ezra, Catherine J. Harmer, Thomas E. Nichols, Najib M. Rahman, Andrea Reinecke, Kyle T.S. Pattinson

**Affiliations:** 1 Wellcome Centre for Integrative Neuroimaging and Nuffield Division of Anaesthetics, Nuffield Department of Clinical Neurosciences, University of Oxford, Oxford, United Kingdom; 2 Department of Psychology, University of Otago, Dunedin, New Zealand; 3 Translational Neuromodeling Unit, University of Zurich and ETH Zurich, Zurich, Switzerland; 4 Department of Psychiatry, Medical Sciences, University of Oxford, Oxford, United Kingdom; 5 Oxford Health NHS Foundation Trust, Warneford Hospital Oxford,; 6 Oxford Big Data Institute, Li Ka Shing Centre for Health Information and Discovery, Nuffield Department of Population Health, University of Oxford, Oxford United Kingdom; 7 Nuffield Department of Medicine, University of Oxford, Oxford, United Kingdom; 8 Oxford NIHR Biomedical Research Centre, Oxford; Chiba Daigaku, JAPAN

## Abstract

Combining traditional therapies such as pulmonary rehabilitation with brain-targeted drugs may offer new therapeutic opportunities for the treatment of chronic breathlessness. Recently, we asked whether D-cycloserine, a partial NMDA-receptor agonist which may enhance behavioural therapies, modifies the relationship between breathlessness related brain activity and breathlessness anxiety over pulmonary rehabilitation. However, whether any changes are supported by alterations to underlying brain structure remains unknown. Here we examine the effect of D-cycloserine over a course of pulmonary rehabilitation on the connectivity between key brain regions associated with the processing of breathlessness anxiety. 72 participants with mild-to-moderate COPD took part in a longitudinal study in parallel to their pulmonary rehabilitation course. Diffusion tensor brain imaging and clinical measures of respiratory function were collected at three time points (before, during and after pulmonary rehabilitation). Participants were assigned to 250mg of D-cycloserine or placebo, which they were administered with on four occasions in a randomised, double-blind procedure. Following the first four sessions of pulmonary rehabilitation (visit 2), during which D-cycloserine was administered, improvements in breathlessness anxiety were linked with increased insula-hippocampal structural connectivity in the D-cycloserine group when compared to the placebo group. No differences were found between the two groups following the completion of the full pulmonary rehabilitation course 4–6 weeks later (visit 3). The action of D-cycloserine on brain connectivity appears to be restricted to within a short time-window of its administration. This temporary boost of the brain connectivity of two key regions associated with the evaluation of how unpleasant an experience is may support the re-evaluation of breathlessness cues, illustrated improvements in breathlessness anxiety.

Trial registration

ClinicalTrials.gov (NCT01985750).

## Introduction

Brain-targeted drugs may offer novel therapeutic opportunities for the treatment of chronic breathlessness. This new field of research builds upon evidence suggesting that active elements of pulmonary rehabilitation, which remains currently the most effective treatment for chronic breathlessness lie, not in improvements to lung function, but in the reappraisal of the sensory experience [1–4]. Furthermore, reappraisal of breathlessness anxiety and intensity as a consequence of pulmonary rehabilitation has been linked to changes in brain activity across a network of attentional and emotional regulation regions, including anterior cingulate cortex and insula cortex [3].

Recently we examined whether the brain-based reappraisal mechanisms of pulmonary rehabilitation could be boosted with the drug D-cycloserine [5,6]. D-cycloserine is a partial N-Methyl-D-Aspartate (NMDA) receptor agonist, which, acting via glutamateric receptors is thought to increase synaptic plasticity and boost the learning processes associated with exposure-based cognitive behavioural therapies (CBT) [7,8]. Considering that 30% of patients undergoing pulmonary rehabilitation derive no clinical benefit [9,10], and health-related benefits typically return to pre-rehabilitation levels 12–18 months after course completion [11,12], therapeutic adjuncts such as D-cycloserine are of considerable clinical relevance to maintaining the effects of pulmonary rehabilitation.

It remains unclear as to whether changes to functional brain activity associated with D-cycloserine administration and subsequent reappraisal of breathlessness anxiety over pulmonary rehabilitation are related to any neuro-plastic structural changes across the brain. Furthermore, while task based functional brain activity has been shown to be somewhat variable across sessions and individuals [13], structural brain changes, which can be measured in terms of volume or connectivity, have good test-retest reliability and may be more sensitive to experience-dependent long-term changes [14]. Such changes may relate to clinical outcomes.

Measures of regional brain volume and connectivity have been shown to change following exposure-based CBT [15,16]. Specifically, posterior and anterior cingulate cortex volume reduction has been linked to symptom improvement and successful cognitive reappraisal following CBT in post-traumatic stress disorder (PTSD) [17,18]. Therefore, such structural changes may become a therapeutic augmentation target for brain-targeted pharmacotherapies. For example, the NMDA receptor blocker ketamine has been shown to block corticosteroid induced volume loss within the hippocampus [19]. Potential targets are not limited to NMDA-related pathways; Lithium has also been shown not only to provide symptomatic relief, but also to increase global grey matter volume in patients with bipolar mood disorder [20], although this may be a feature of altered image contrast [21].

Given that emotional learning is associated with structural changes within grey [22] and white matter [23] and D-cycloserine is thought to boost the learning-related process of exposure-based CBT via increased synaptic plasticity, we hypothesised that D-cycloserine would modify brain connectivity within/between five key brain regions of interest. These regions were: anterior insular cortex, posterior insular cortex, anterior cingulate cortex, amygdala and hippocampus. Both the amygdala and hippocampus have been identified by animal studies as key target sites for the modulation of emotional learning by D-cycloserine [24,25], while changes to activity within insula and anterior cingulate cortex have been shown to correlate with improvements across pulmonary rehabilitation [3,6].

## Methods

An overview of the methodology is presented here, and extended details can be found within supplementary materials. The study and statistical analysis plan of the primary analyses were pre-registered on clinicaltrials.gov (ID: NCT01985750) prior to unblinding. The authors confirm that all ongoing and related trials for this intervention are registered. This was a novel and secondary analysis of data from a longitudinal experimental medicines study of patients with COPD over a course of pulmonary rehabilitation. Data from this study were first published in a characterisation of baseline patient clusters [26] and subsequently in an investigation of the effect of D-cycloserine on functional brain activity across pulmonary rehabilitation [5]. The analysis conducted here explores the brain’s structure rather than function, is novel and not previously reported.

**Fig 1:**
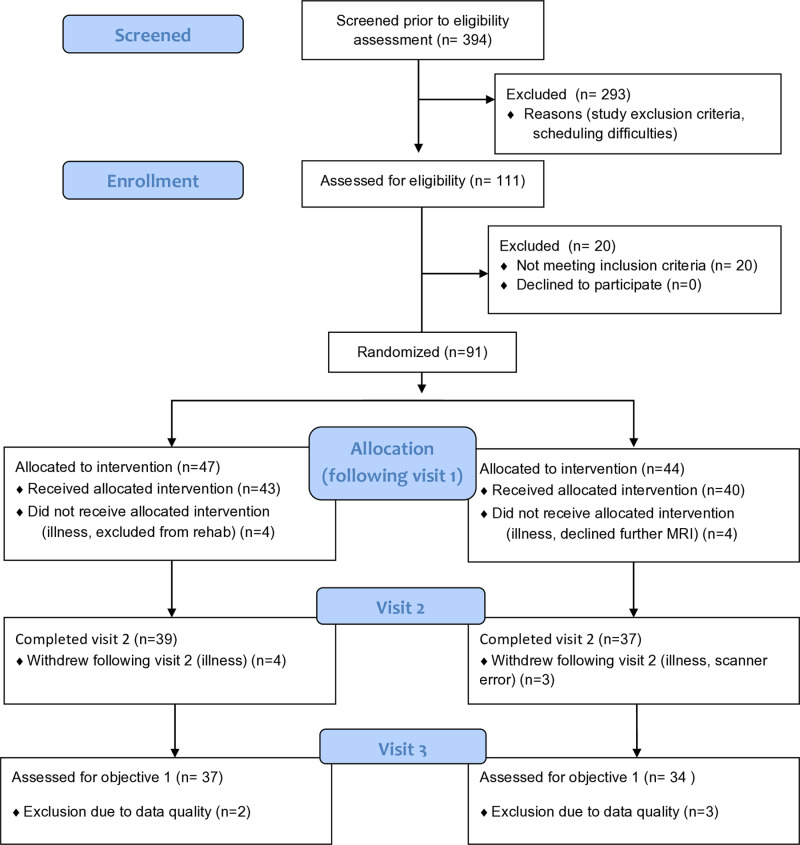
Consort diagram.

72 participants (18 female, median age 71 years (46–85 years)) (Table 1, Fig 1.) with COPD were recruited immediately prior to their enrolment in a National Health Service-prescribed course of pulmonary rehabilitation. Written informed consent was obtained from all participants prior to the start of the study. The dates of first and last recruitment were 12/11/2013 and 30/04/2018 with final follow up 28/06/2018. Study approval was granted by South Central Oxford REC B (Ref: 118784, Ethics number: 12/SC/0713). Study inclusion criteria were: a diagnosis of COPD and admittance to pulmonary rehabilitation. Exclusion criteria were: inadequate understanding of verbal and written English, significant cardiac, psychiatric (including depression under tertiary care) or metabolic disease (including insulin-controlled diabetes), stroke, contraindications to either D-cycloserine (including alcoholism) or magnetic resonance imaging (MRI), epilepsy, claustrophobia, regular therapy with opioid analgesics or home oxygen therapy.

**Table 1:** Demographic information. From the 72 participants who completed all three study visits. Variance is expression either in terms of standard deviation (SD) or interquartile range (IQR) depending on the normality of the underlying data distribution. BMI = Body Mass Index, MRC = Medical Research Council. SpO2% = Peripheral Oxygen saturation, expressed as a percentage. FEV = Forced Expiratory Volume. FVC = Forced Vital Capacity. Also listed with prevalence in brackets are recorded comorbidities ordered by frequency.

	Visit 1 (Pre-rehabilitation)	
	D-cycloserine (N = 37)	Placebo (N = 34)
Age (median years/ range)	**71.0/ (47-81)** **34.0/ (25.6)** **8.0 (17.0)**	**71.5/ (46-85)**
Smoking pack-years (years/ IQR)		**30.0/ (30.0)**
Duration of breathlessness (years/ IQR)		**9.5 (10.0)**
Total exacerbations (number/ IQR)	**0.0 (1.0)** **27.3 ± 6.5** **3.0 (1)** **95/ (3.3)** **80.8 ± 13.4** **0.53/ (0.17)**	**1.0 (2.3)**
BMI (kg.m-2 ± SD)		**26.9 ± 5.7**
MRC breathlessness scale (IQR)		**2.5 (1)**
Resting SpO_2_% (IQR)		**95/ (3.0)**
Resting heart rate (beats.min-1 ± SD)		**80.8 ± 14.9**
FEV1/FVC (IQR)		**0.56/ (0.13)**
FEV1% Predicted (IQR)	**51.7 (31.2)**	**66.8 (25.2)**
GOLD		
1 (A/B/C/D)	**10 (0/10/0/0)**	**6 (1/5/0/0)**
2 (A/B/C/D)	**14 (3/11/0/0)**	**14 (0/14/0/0)**
3 (A/B/C/D)	**12 (0/0/0/12)**	**11 (0/0/0/11)**
4 (A/B/C/D)	**1 (0/0/0/1)**	**3 (0/0/0/3)**
	Comorbidities (frequency)	
	Asthma (14)	Asthma (11)
	Hypertension (13)	Hypertension (11)
	Gastro-oesophageal reflux (10)	Gastro-oesophageal reflux (12)
	Swelling of both ankles (11)	Swelling of both ankles (8)
	Surgery to the chest (6)	Surgery to the chest (7)
	Depression (6)	Depression (2)
	Diabetes (3)	Diabetes (6)
	Heart attack (4)	Heart attack (5)
	Bronchiectasis (3)	Bronchiectasis (4)
	Osteoporosis (2)	Osteoporosis (4)
	Arrhythmia (3)	Arrhythmia (4)
	Inflammatory bowel disease (2)	Inflammatory bowel disease (3)
	Peptic ulcer (3)	Peptic ulcer (2)
	Heart failure (1)	Heart failure (1)
	Tuberculosis (1)	Neuromuscular weakness (2)

### Study drug.

Participants were randomised in a double-blinded procedure to receive either 250mg oral D-cycloserine or a matched placebo, administered by the study nurse 30 minutes prior to the onset of their first four pulmonary rehabilitation sessions. Study participants, investigators and those performing the analysis were blinded to the treatment allocation. Both D-cycloserine and placebo were over-encapsulated to appear identical. Full study drug description, randomisation protocol and minimisation criteria can be found within the supplementary materials.

### Sample size

At the time of study inception (and to a large extent still to date), the literature regarding D-cycloserine’s effects on functional brain activity, for which this trial was powered was very limited. Therefore, to calculate the sample sizes required for this study we first took into account the described effects of D-cycloserine in clinical studies of augmentation for cognitive behavioural therapy for anxiety disorders. As this is a behavioural outcome, we expected this to have sufficient power to detect change in functional brain activity signalling which was the primary outcome of the trial and is reported elsewhere [5]. Full details regarding the calculation are reported withing supplementary materials.

#### Study visit protocol.

Following telephone screening, participants were invited to attend their first research session (baseline) at the John Radcliffe Hospital Oxford prior to starting pulmonary rehabilitation. A second study visit took place following the fourth pulmonary rehabilitation session but before the sixth session. Participants completed the remainder of their pulmonary rehabilitation course before attending a third study session (Figure in S1) that occurred as close to the termination of pulmonary rehabilitation as possible and always within two weeks.

#### Patient and public involvement.

Participants were involved in study dissemination activities. The research team visited pulmonary rehabilitation centres to present primary study results, answer questions and contacted participants who had indicated they would be interested to hear updates of study progress.

#### Pulmonary rehabilitation.

Pulmonary rehabilitation courses were run by either Oxford Health NHS Foundation Trust, West Berkshire NHS Foundation Trust, or Milton Keynes University Hospitals NHS Trust. The full course ran for 6 weeks with two sessions per week including an hour of exercises and an hour of education, as part of a standard pulmonary rehabilitation programme. Full details of pulmonary rehabilitation can be found within supplementary materials.

#### Physiological measures.

Spirometry and two Modified Shuttle Walk Tests (MSWT) were collected using standard protocols [27,28]. Participant height and weight were recorded at each visit. Oxygen saturations and heart rate were measured with pulse oximetry and were collected at rest and following the MSWT.

#### MRI measures.

*Image acquisition* Magnetic resonance imaging of the brain was carried out using a Siemens 3T MAGNETOM Trio. A T1-weighted (MPRAGE) structural scan (voxel size: 1 x 1 x 1 mm) was collected and used for registration purposes. Diffusion weighted images were acquired in the transverse plane using an echo planar imaging sequence (2 acquisitions of 64 directions with 4 non-diffusion weighted images, *b-*value 1,500 s mm^-2^ voxel size 2 x 2 x 2 mm, 64 slices)

A T2*-weighted, gradient echo planar image (EPI) scan sequence (voxel size: 3 x 3 x 3 mm), TR, 3000ms; TE 30ms was used to collect FMRI data. FMRI data are reported elsewhere [5,6,29].

#### Pre-processing.

**Diffusion MRI**: Data were pre-processed following standard steps within FMRIB’s diffusion toolbox (FDT) [30]. First non-brain tissue was removed, and susceptibility-induced distortions and eddy currents were corrected using the FSL tools topup and eddy [31,32]. A diffusion tensor was fitted for each voxel using the FSL tool dtifit. A ball and stick probability model was fitted to the data using the FSL bool BEDPOSTX prior to estimating the tract paths.

**Fig 2:**
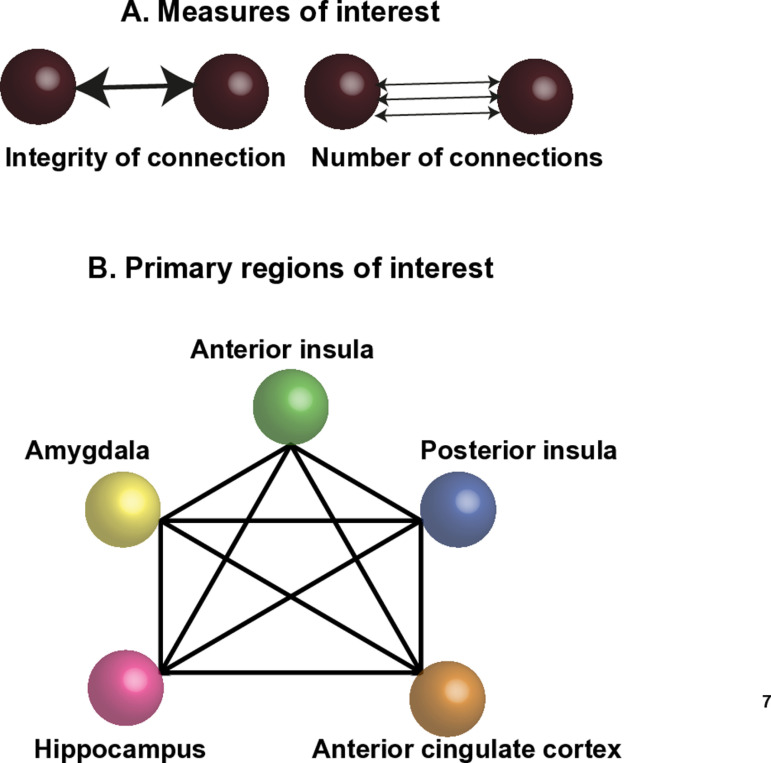
Connectivity illustration. The two measures of interest – the integrity of the regional connections (FA) and the number of connections (streamlines). Schematic representation of the five key regions of interest and their connectivity.

**Connectivity analysis**: Probabilistic tractography was run on the output of the probabilistic model with seeds specified as each of the regions of interest (using the FSL tool PROBTRACKX). A 5x5 matrix of the 5 regions of interest was included, indicating the seed to target masks. This generated values of the total number of streamlines between each region of interest for each subject (Fig 2.).

**Structural integrity analysis - Fractional Anisotropy**: FSL’s standard Tract Based Spatial Statistics (TBSS) pipeline [31,33] was used to estimate the integrity of connections, termed fractional anisotropy (FA). All subjects’ FA data were aligned into a common space using the nonlinear registration tool FNIRT [34], which uses a b-spline representation of the registration warp field [35]. Next, the mean FA image was created and thinned to create a mean FA skeleton which represents the centres of all tracts common to the group. Each subject’s aligned FA data was then projected onto this skeleton and the resulting data fed into voxelwise cross-subject statistics. An average streamline density map was created by adding together all single subjects streamline density maps, created by FSL’s FDT toolbox.

### Group level analysis

#### Primary analysis – Focused region of interest analysis.

Only complete datasets were included. For each patient, the following metrics were extracted from each of the five regions of interest at visits two and three having accounted for baseline activity (Figure in S1, panel A).

1. Region to region connectivity (number of streamlines) calculated using probabilistic tractography
2. Regional Fractional Anisotropy (FA), a measure of structural integrity

To test for an overall drug effect, values relating to regional connectivity and integrity were entered into independent linear mixed effects models where they were adjusted for age and gender to account for differences in brain volume. Additionally, connectivity or integrity (FA) at visit one (depending on the metric) was also accounted for within the model. Permutation testing was performed with threshold free cluster enhancement (TFCE) (a non-parametric test) [36] using FSL’s Randomise tool [37] at family wise error corrected p < 0.05. This corrected results for multiple comparisons. The process was then repeated separately for data collected at visit three. To additionally test whether a drug effect was related to improvements in reported breathlessness anxiety, models were re-run to include breathlessness anxiety as an additional model term. Models were programmed using the lme4 function within R version 3.6.1 (2019-07-05).

## Results

In a simple comparison of drug versus placebo, no differences were found in brain connectivity. However, accounting for improvements in breathlessness anxiety, which in this instance is a surrogate marker for the effectiveness of pulmonary rehabilitation revealed a difference between placebo and D-cycloserine groups.

Four participants were excluded due to incomplete MRI datasets. There was no significant overall effect of D-cycloserine on mean brain connectivity within the five key regions of interest (anterior cingulate, anterior insula cortex, amygdala, hippocampus or posterior insula cortex) compared to placebo (S3 Table) at visit 2 or visit 3. No differences were found in changes to reported breathlessness anxiety between D-cycloserine and placebo groups at visits two or three (S1 Table).

**Fig 3:**
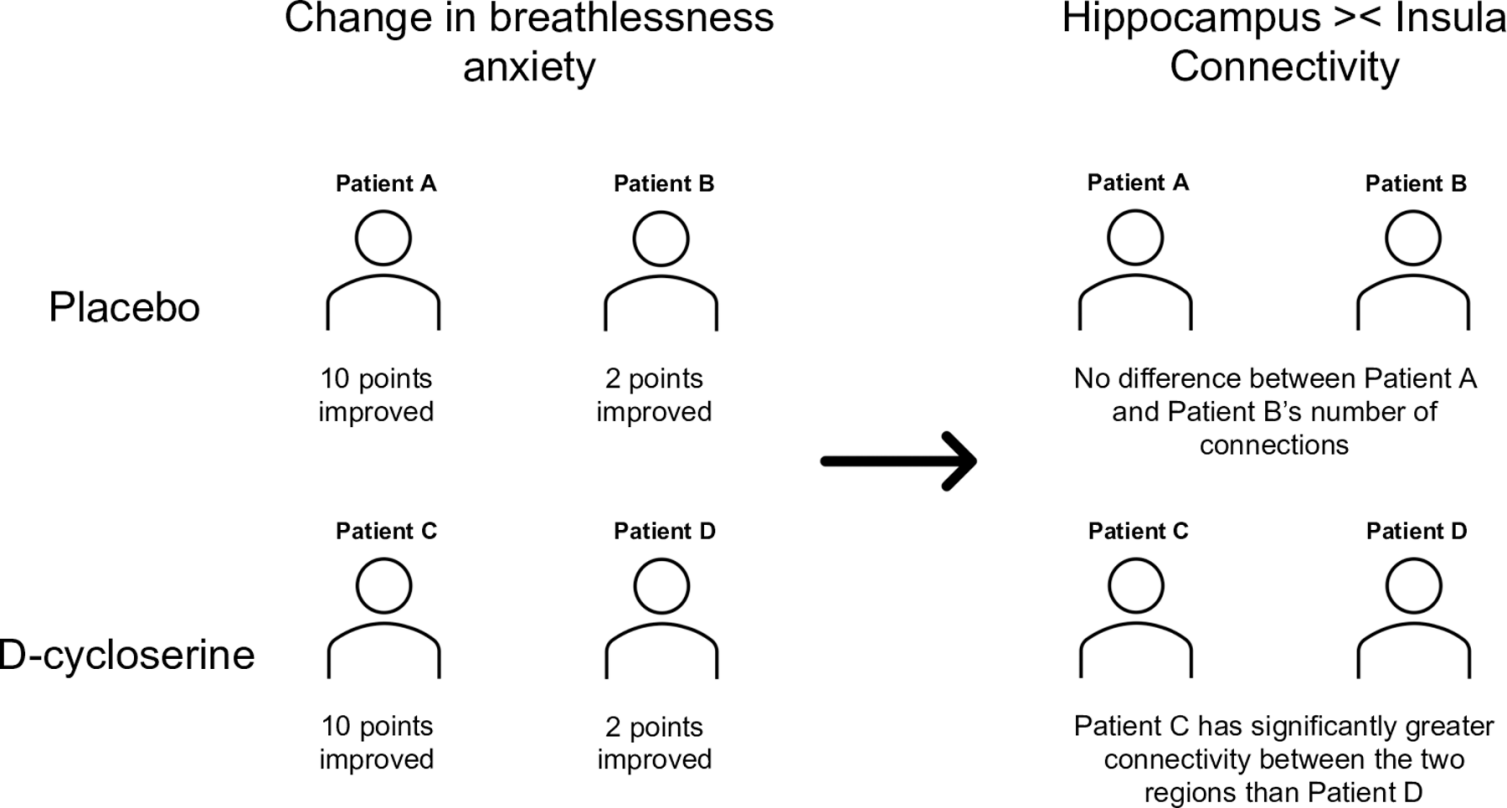
Illustration of key finding. A schematic illustrating the relationship between drug group, changes to breathlessness anxiety and brain connectivity with illustrative numbers.

**Fig 4:**
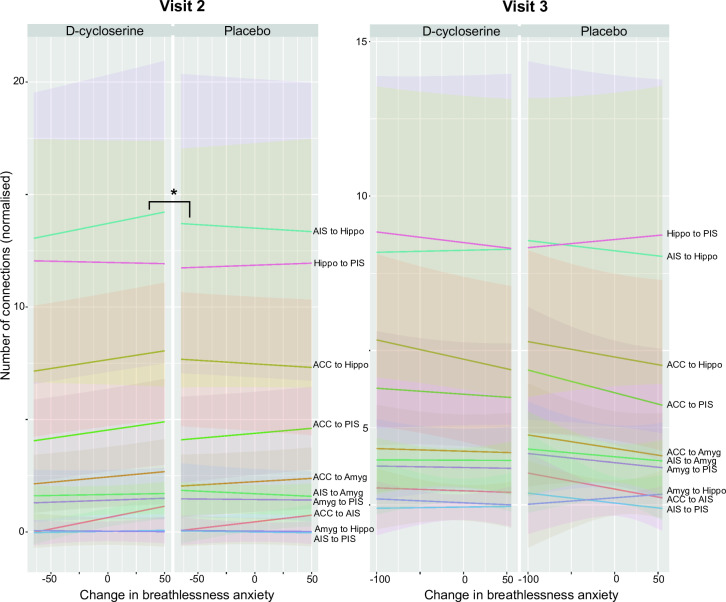
Interaction plots. Plots of interaction between brain region, change in breathlessness anxiety and drug/placebo.

Participants in the D-cycloserine group who gave lower ratings for breathless anxiety at visit 2 had a correspondingly greater number of connections between the two regions than those whose breathlessness anxiety did not change as much (p = 0.04, corrected family wise error rate 5%) (S3 Table) (Figs 2–4). This relationship was not observed in the placebo group, and was no longer observed at visit three, following the completion of pulmonary rehabilitation 4–6 weeks later, during which time no further D-cycloserine administrations were given. No differences between D-cycloserine and placebo groups were observed in fractional anisotropy, a measure of the integrity of existing connections at visit 2 or visit 3. All statistical tests were corrected for multiple comparisons using a family wise error rate 5%.

## Discussion

Our results showed a relationship between brain connectivity, improvements in breathlessness anxiety and D-cycloserine.

After four sessions of rehabilitation with concurrent D-cycloserine (Visit 2), participants who received D-cycloserine and reported lower breathlessness anxiety had greater connectivity between anterior insula and hippocampus compared to the placebo group. Following the completion of pulmonary rehabilitation with no further pharmacotherapy (Visit 3), this effect was no longer observed.

Pulmonary rehabilitation, which draws on elements of exposure-based cognitive behavioural therapy, provides symptomatic relief for around 70% of participants [3,4,38]. However, for most patients, health-related benefits return to pre-rehabilitation levels 12–18 months after course completion [11,12]. This has led to research exploring whether the therapeutic efficacy of pulmonary rehabilitation could be boosted or extended.

It is well established that emotional learning processes are associated with changes to brain structure [22, 39], while brain targeted drugs have also been shown to affect both structural and functional connectivity [19,40], as well as regional volume [20]. Here we identified no overall effect of D-cycloserine on brain connectivity. Instead, in keeping with our previous findings, which highlighted the need to take into account a surrogate marker of rehabilitation success [6] we found a greater number of connections between anterior insula and hippocampus for people who reported a greater reduction in breathlessness anxiety after the first four sessions of pulmonary rehabilitation with concurrent D-cycloserine administration. By the end of pulmonary rehabilitation (visit three) the difference between placebo and D-cycloserine groups was no longer observed, suggesting this effect to be related temporally to the D-cycloserine administration period or its interaction with the initiation of pulmonary rehabilitation. It is possible that the short-term relationship between connectivity and change in breathlessness anxiety within two regions associated with internal networks of bodily perception (interoception) may have then facilitated the functional changes observed across a network of emotional salience regions after the completion of pulmonary rehabilitation [5,6].

The difference in effect of D-cycloserine observed in this study at visit two (brain structure) and in a previously published work after the completion of pulmonary rehabilitation (brain activity) [6] fits with recent meta-analysis of D-cycloserine action [7] and offers potential insight into the nature of breathlessness reappraisal. It has been suggested that the somewhat unpredictable success rate of D-cycloserine in exposure-based therapies could be explained by whether the target for learning relies on high- or low-order conditioned responses. Lower-order responses are automatic, such as the startle reflex, whereas higher-order responses describe a reaction learned over time following repeated exposures. Lower-order responses may be easier targets for D-cycloserine’s NMDA-receptor action via the limbic system, which include anterior insula – hippocampus connections, compared to higher-order learned responses embedded within frontal networks [7]. Certainly, breathlessness can be considered as both a high and low order conditioned response. While the experience of breathlessness includes autonomic and implicit processing, for many the deeply embedded and individual learned associations will be higher-order and more resilient. Here, we observed a measurable difference in anterior insula and hippocampus connectivity linked to the revaluation of breathlessness cues in the D-cycloserine group. In people for whom pulmonary rehabilitation was successful, this effect may then have translated over the course of pulmonary rehabilitation to tackle the more deeply embedded fearful associations of breathlessness, resulting in broader functional changes across higher-order brain regions [6].

### Limitations and future directions

These preliminary results suggest that D-cycloserine action may relate to changes in breathlessness anxiety. While we were able to use changes to breathlessness anxiety as a surrogate marker for pulmonary rehabilitation effectiveness, future studies should consider collecting post pulmonary rehabilitation session reports for model inclusion. Questions remain outstanding as to why this effect was seen at visit two (after four sessions of rehabilitation) but not at visit three (after the completion of pulmonary rehabilitation). It is also important to highlight that the insula-hippocampus connectivity was one of several connections assessed. Given the statistical significance of the result is relatively weak (p = 0.04), it naturally raises questions relating to false positives, however, our statistical approach which included stringent correction for multiple comparisons largely mitigated for this risk. Additionally, pulmonary rehabilitation is a strong behavioural intervention, and recent literature has suggested that D-cycloserine action is curtailed near the therapeutic ceiling [41]. Thus, pairing a strong drug with a weaker behavioural therapy may provide more scope for mechanistic investigations of overall group differences. Future studies should consider accounting for individual differences with more formally specified outcome measures. In addition, at study inception there was limited information available to support power calculations. Calculations of sample sizes took into account the described effects of D-cycloserine in clinical studies of augmentation for cognitive behavioural therapy for anxiety disorders. It is therefore possible that a larger sample size may address some of the low statistical power observed in this study.

## Conclusions

We have provided early evidence suggesting that D-cycloserine may have a temporary effect on brain structure. It is possible that these changes may be a precursor for changes to brain activity and the reappraisal of emotional responses to breathlessness related cues. This also highlights reinforcement learning as a potential mechanism and therefore also a target in the effectiveness of pulmonary rehabilitation. This effect requires further study both to validate the existing results and to further unpick the mechanism of action.

## Supporting information

S1 Fig CONSORT-2010-Checklist-MS-Word (DOCX)

S1-3 Tables Significance of overall group effect of D-cycloserine on the integrity of connections. (DOCX)
