# Supplementary material for: The effect of D-cycloserine on brain connectivity over a course of pulmonary rehabilitation – A randomised control trial with neuroimaging endpoints
Source: PLoS One. 2025 Jun 2;20(6):e0323213. doi: 10.1371/journal.pone.0323213 (PMC12129347; doi:10.1371/journal.pone.0323213)
Supplement: S1-3 Tables — (DOCX) [file pone.0323213.s002.docx]

**The effect of D-cycloserine on brain connectivity over a course of pulmonary rehabilitation**

Sarah L. Finnegan^1^ ([sarah.finnegan@ndcn.ox.ac.uk](mailto:sarah.finnegan@ndcn.ox.ac.uk))

Olivia K. Harrison^2^

Martyn Ezra^1^

Catherine J. Harmer ^3,4^

Thomas E. Nichols ^5,1^

Najib M. Rahman^6,7^

Andrea Reinecke ^3,4^

Kyle T.S. Pattinson^1^ ([kyle.pattinson@nda.ox.ac.uk](mailto:kyle.pattinson@nda.ox.ac.uk))

^1^ Wellcome Centre for Integrative Neuroimaging and Nuffield Division of Anaesthetics, Nuffield Department of Clinical Neurosciences, University of Oxford, Oxford, UK.

^2^. School of Psychology, University of Otago, Dunedin, New Zealand

^3^. Department of Psychiatry, Medical Sciences, University of Oxford, Oxford, UK;

^4^. Oxford Health NHS Foundation Trust, Warneford Hospital Oxford

^5^. Oxford Big Data Institute, Li Ka Shing Centre for Health Information and Discovery, Nuffield Department of Population Health, University of Oxford, Oxford OX3 7LF UK

^6^ Nuffield Department of Medicine, University of Oxford, Oxford, UK

^7^ Oxford NIHR Biomedical Research Centre, Oxford OX3 7JX

**Online Data Supplement**

**Study Drug**

Study drugs were purchased from Ipswich Hospital Pharmacy Manufacturing Unit, Heath Road, Ipswich IP4 5PD, Tel: 01473 703603. Participants were administered with 250mg of D-cycloserine. While 50mg remains the most common dosage for D-cycloserine, there is no evidence to suggest that 50mg is more effective than higher dosages [1]. Furthermore, work conducted in healthy volunteers suggests that hippocampal learning only occurred at 250mg dose and not at 50mg [2]. The efficacy of 250mg dosage on brain plasticity is supported by more recent work [3] which found changes to amygdala reactivity after a single administration of D-cycloserine.

**Randomisation Procedure**

Once the participant gave written consent to the trial and completed the MRI scan, a member of the team submitted a randomisation form, entering eligibility criteria and minimisation factors. Allocation to active or placebo capsules, which were both over-encapsulated to appear identical, was carried out by Sealed Envelope Randomisation Services (Sealed Envelope Ltd, Concorde House, Grenville Place, London NW7 3SA). The randomisation number was then provided to the Oxford Respiratory Trials Unit who dispensed the drug/placebo. Minimisation factors were as follows:

1. Centre

2. MRC grade

3. Diabetes

4. Antidepressant

5. Age at which the participant completed full time education

6. Previous rehabilitation

Randomisation codes were held by Sealed Envelope until study completion, after which at the first stage of unblinding an independent researcher provided study researchers with a coded binarised system for analysis. Researchers remained blinded to group identity until analysis was completed.

**Sample Size**

At the time of study inception (and to a large extent still to date), the literature regarding D-cycloserine’s effects on functional brain activity is very limited. Therefore, in order to calculate the sample sizes required for this study we first took into account the described effects of D-cycloserine in clinical studies of augmentation for cognitive behavioural therapy for anxiety disorders, where effect sizes of up to 1.06 have been reported (although more commonly 0.4 to 0.7) [4-7]**.** The most relevant paper (on treatment of snake phobia [8]**)** demonstrated that effects observed with neuroimaging were more sensitive than behavioural effects, therefore we believe that powering for a behavioural outcome measure (breathlessness-anxiety) provided a safe margin and was likely to be sufficiently conservative to detect our measures of interest. This was particularly the case as compared to the relatively blunt nature of behavioural data collection, functional neuroimaging carries considerably more specificity and statistical power. The study was not therefore specifically powered to investigate the clinical effects of D-cycloserine. In our previous study we observed an 11% (SD15% around the mean) improvement in breathlessness-related anxiety, measured with our FMRI word task (pre-treatment mean score 38%, post treatment mean score 27%, difference 11%, SD15% around the mean) [9]. Making a conservative assumption, we estimated that D-cycloserine augments this response with an effect size of 0.4. Assuming a similar coefficient of variation we anticipated an 18% (SD24%) improvement in breathlessness-anxiety (i.e. pre-treatment mean score 38%, post treatment mean score 20%, difference 18%, SD of difference 24%). Assuming α=0.05 and power 0.80, then we estimated a sample size of 36 in each group randomised 1:1. As this is a behavioural outcome, we expected this to have sufficient power to detect change in BOLD signalling which was the primary outcome of the trial and is reported elsewhere [10].

**Pulmonary rehabilitation details**

Pulmonary rehabilitation was delivered by an experienced community pulmonary rehabilitation team. The full course ran for 6 weeks, with two sessions per week including an hour of exercises and an hour of education, as part of a standard pulmonary rehabilitation programme. Patients had been referred to pulmonary rehabilitation as part of their standard management. As pulmonary rehabilitation courses vary in duration and content we briefly describe the course that the patients in this study undertook. The Oxfordshire pulmonary rehabilitation programme is a cohort course which consists of two- hour sessions performed twice weekly for six weeks in an outpatient setting. This was run by Oxford Health NHS Foundation Trust. The Reading pulmonary rehabilitation programme differed in that it enrolled patients as part of a rolling intake rather than as a cohort. This was run by Berkshire Healthcare NHS. The Milton Keynes pulmonary rehabilitation programme enrolled patients as a cohort and took place within the hospital. This was run by Milton Keynes University Hospital NHS Foundation Trust. Content of the three courses was very similar.

Outpatient settings were non-medical facilities with the appropriate exercise equipment available, e.g. sports halls. Each session consisted of one hour of exercise (under supervision) and one hour of education.

Exercise sessions included both aerobic and strength exercises, tailored to the individual's ability. Aerobic exercises could include step-ups, walking (on the spot or treadmill) and exercising on a cycle ergometer. Strength exercises were conducted in sets (usually 3) of ten, and included sit-to-stand exercises, biceps curls, upright row and leg extensions.

Education sessions included items such as ‘introduction to rehabilitation’, ‘management of breathlessness’, ‘airway clearance’, ‘understanding your lung condition’, ‘home exercises’, medicine management’, ‘staying healthy’, ‘stress and relaxation’, ‘pacing and energy conservation’, ‘smoking cessation’, ‘continuing support’, ‘sexual function’ and ‘advanced care plans’. Supervised modified shuttle walking tests were used to measure patients’ improvement throughout the programme.

**Physiological Measures**

A trained respiratory nurse collected spirometry measures of FEV_1_ and FVC using Association for Respiratory Technology and Physiology standards [11]. Participants performed two modified incremental shuttle walk tests (MSWT) [12], and heart rate and oxygen saturations (SpO_2_) were measured immediately before the MSWT and subsequently every minute until 10 minutes post-exercise (or until participants returned to their baseline state) using a fingertip pulse oximeter (Go_2_; Nonin Medical Inc). Before and after the MWST participants also rated their breathlessness on a modified Borg scale [13]. In a MWST participants must walk between and around two cones, placed 10m apart in time to a set of auditory beeps played from a laptop. Initially the speed of beep repetition is slow, but the participant must increase their walking speed each minute in order to reach the cone before the next beep. Participants continue to walk (or run) until they are too breathless to continue, at which point the total distance walked is recorded.

**MRI Acquisition**

Prior to each MRI session participants were screened for standard MRI contraindications including metal in or about their person, epilepsy and claustrophobia.

**Image acquisition:**

Hardware: A Tim System (Siemens Healthcare GmbH) 12-channel head coil.

T1 sequence parameters: TR, 2040ms; TE, 4.68ms; voxel size, 1 x 1 x 1 mm; FOV, 200mm; flip angle, 8°; inversion time, 900ms; bandwidth 130 Hz/Px).

**Region of interest extraction**

The five bilateral regions of interest (ROI) were anterior insula cortex, posterior insula cortex, anterior cingulate cortex, amygdala and hippocampus. Seed voxels for each region of interest were identified as the peak voxel co-ordinates (Supplementary table 2) responding to breathlessness word-cues published by Herigstad et al 2017 [9] within the boundaries of each region of interest identified by standard atlas maps. The seed voxels were expanded to include the surrounding voxels within a 5 mm radius.

Left and right masks of bilateral regions of interests (anterior insula, posterior insula, anterior cingulate, amygdala and hippocampus) were added together to form one mask for each region of interest. Following registration each mask was re-thresholded at 40% probability to avoid interpolation errors before being binarised.

***S1 Table.*** *MNI coordinates for region of interest seeds*

| Region of interest | Hemisphere | x | y | z |
| --- | --- | --- | --- | --- |
| Anterior insula cortex | Left | -31 | 7 | -14 |
|  | Right | 38 | 13 | -10 |
| Posterior insula cortex | Left | -31 | 7 | -15 |
|  | Right | 37 | 10 | -12 |
| Anterior cingulate cortex |  | -5 | 34 | -3 |
| Amygdala | Left | -19 | -7 | -21 |
|  | Right | 19 | -8 | -18 |
| Hippocampus | Left | -22 | -12 | -26 |
|  | Right | 20 | -9 | -19 |

**Network mask**

A second network mask region of interest was created from the 5 core regions of interest outlined above and an additional 11 regions defined by standard anatomical atlas maps (Harvard-Oxford Atlas and Destrieux’ cortical atlas) (Fig S1). A 40% probability threshold was applied to each region, before they were combined along with the original 5 regions into one network mask. This network mask was then registered to each individual before being re-thresholded at 40% probability to avoid interpolation errors and binarized. Combining the 16 regions into a single mask enabled us to appropriately correct for multiple comparisons.

**S1. Fig. Regions of interest.** Panel A highlights the 5 key regions of interest while Panel B shows the expanded region of interest map.

**Analysis**

**Volumetric MRI Pre-processing**

Structural data was analysed with FSL-VBM [14], carried out with FSL tools [15]. Firstly, the T1 images were corrected for White Matter Hypo-intensities (WMH), which are commonly associated with COPD and may result in misclassification of white matter into grey matter. Correction was carried out using a lesion probability map, created by FSL’s Brain Intensity AbNormality Classification Algorithm (BIANCA) [29]. Each subjects’ T1 image was skull stripped and segmented (FSL FAST) [30] before the grey matter was registered non-linearly to the MNI152 brain template [16]. A study specific template was created from the average of the transformed grey matter images. All native GM images were then non-linearly registered to this study-specific template and “modulated” to correct for local expansion and contraction due to non-linear components of the spatial transformation. The modulated GM images were smoothed with an isotropic Gaussian kernel with a sigma of 3 mm (~7 mm Full Width Half Maximum (FWHM)) prior to statistical analyses.

**S2 Table.** **Significance of overall group effect of D-cycloserine on the integrity of connections**. Measured as fractional anisotropy, and number of connections between the five key regions of interest at visits two and three, having accounted for these measures at visit one. Significance is corrected for multiple comparisons and reported as Family Wise Error (p<0.05) corrected p-values of the difference.

|  |  | | **Visit two** | | | **Visit three** | | |
| --- | --- | --- | --- | --- | --- | --- | --- | --- |
| **Region of interest** | | **Connections** | **Estimate** | **Std. Error** | **p-value** | **Estimate** | **Std. Error** | **p-value** |
| Anterior cingulate – Anterior insula | | Integrity | 0.03 | 0.13 | 1.00 | 0.00 | 0.17 | *1.00* |
|  |  | Number | -0.13 | 0.11 | 0.84 | 0.03 | 0.13 | *1.00* |
| Anterior cingulate – Amygdala | | Integrity | -0.12 | 0.14 | 0.98 | 0.26 | 0.17 | 0.70 |
|  |  | Number | -0.19 | 0.12 | 0.59 | 0.07 | 0.13 | *0.98* |
| Anterior cingulate – Hippocampus | | Integrity | 0.07 | 0.14 | 1.00 | 0.35 | 0.18 | 0.34 |
|  |  | Number | -0.18 | 0.12 | 0.62 | 0.01 | 0.13 | *1.00* |
| Anterior cingulate – Posterior insula | | Integrity | 0.06 | 0.14 | 1.00 | -0.14 | 0.17 | *0.97* |
|  |  | Number | -0.10 | 0.11 | 0.89 | 0.00 | 0.13 | *1.00* |
| Anterior insula - Amygdala | | Integrity | 0.10 | 0.14 | 0.99 | -0.09 | 0.17 | 0.98 |
|  |  | Number | 0.03 | 0.11 | 1.00 | 0.10 | 0.13 | *0.94* |
| Amygdala – Posterior insula | | Integrity | 0.06 | 0.14 | 1.00 | 0.12 | 0.17 | 0.97 |
|  |  | Number | 0.04 | 0.11 | 1.00 | 0.14 | 0.13 | *0.85* |
| Anterior insula - Hippocampus | | Integrity | -0.00 | 0.14 | 1.00 | 0.09 | 0.18 | 0.98 |
|  |  | Number | -0.20 | 0.12 | 0.54 | -0.03 | 0.13 | *1.00* |
| Amygdala – Hippocampus | | Integrity | 0.02 | 0.14 | 1.00 | 0.13 | 0.17 | 0.97 |
|  |  | Number | -0.03 | 0.11 | 1.00 | 0.12 | 0.13 | *0.91* |
| Anterior insula – Posterior insula | | Integrity | 0.07 | 0.14 | 1.00 | 0.27 | 0.18 | 0.69 |
|  |  | Number | 0.00 | 0.12 | 1.00 | 0.16 | 0.13 | *0.77* |
| Hippocampus – Posterior insula | | Integrity | -0.30 | 0.14 | 0.24 | -0.08 | 0.17 | 0.98 |
|  |  | Number | -0.12 | 0.11 | 0.85 | 0.09 | 0.13 | 0.95 |

**S3 Table.** **Significance of the relationship between D-cycloserine and breathlessness anxiety (wA) on the integrity of connections.** Measured as fractional anisotropy and number of connections between the five key regions of interest at visits two and three, having accounted for these measures at visit one. Significance is corrected for multiple comparisons and reported as Family Wise Error (p<0.05) corrected p-values of the difference.

|  |  | | **Visit two** | | | **Visit three** | | |
| --- | --- | --- | --- | --- | --- | --- | --- | --- |
| **Region of interest** | | **Connections** | **Estimate** | **Std. Error** | **p-value** | **Estimate** | **Std. Error** | **p-value** |
| Anterior cingulate – Anterior insula | | Integrity | -0.00 | 0.01 | 0.99 | -4.94e-06 | 9.87e-03 | 1.00 |
|  |  | Number | -4.2e-03 | 4.56 e-03 | *0.96* | -0.00 | 0.01 | 1.00 |
| Anterior cingulate – Amygdala | | Integrity | -0.01 | 0.01 | 0.89 | -7.35e-03 | 9.89e-03 | 0.98 |
|  |  | Number | -1.8e-03 | 4.60 e-03 | *0.99* | -0.00 | 0.01 | 1.00 |
| Anterior cingulate – Hippocampus | | Integrity | -0.00 | 0.01 | 0.99 | 1.32e-02 | 9.86e-03 | 0.84 |
|  |  | Number | -1.1e-02 | 4.55 e-03 | *0.12* | 0.01 | 0.01 | 1.00 |
| Anterior cingulate – Posterior insula | | Integrity | -0.01 | 0.01 | 0.90 | 5.06e-03 | 9.87e-03 | 0.99 |
|  |  | Number | -2.9e-03 | 4.56 e-03 | *0.99* | -0.01 | 0.01 | 0.98 |
| Anterior insula - Amygdala | | Integrity | -0.01 | 0.01 | 0.50 | -9.97e-03 | 9.98e-03 | 0.96 |
|  |  | Number | -3.2e-03 | 4.54 e-03 | *0.99* | -0.00 | 0.01 | 1.00 |
| Amygdala – Posterior insula | | Integrity | -0.01 | 0.01 | 0.75 | -2.25e-04 | 9.90e-03 | 1.00 |
|  |  | Number | -2.2e-03 | 4.56 e-03 | *0.99* | -0.00 | 0.01 | 1.00 |
| Anterior insula - Hippocampus | | Integrity | -0.00 | 0.01 | 0.99 | 7.53e-03 | 9.86e-03 | 0.98 |
|  |  | Number | -1.4e-02 | 4.69 e-03 | ***0.04**** | -0.00 | 0.01 | 1.00 |
| Amygdala – Hippocampus | | Integrity | -0.00 | 0.01 | 0.99 | 8.59e-03 | 9.90e-03 | 0.98 |
|  |  | Number | 7.2e-05 | 4.56 e-03 | *0.99* | 0.00 | 0.01 | 1.00 |
| Anterior insula – Posterior insula | | Integrity | -0.00 | 0.01 | 0.99 | -5.20e-03 | 9.88e-03 | 0.99 |
|  |  | Number | -1.6e-03 | 4.57 e-03 | *0.99* | -0.00 | 0.01 | 1.00 |
| Hippocampus – Posterior insula | | Integrity | 0.01 | 0.01 | 0.83 | 5.46e-03 | 9.86e-03 | 0.99 |
|  |  | Number | -2.9e-03 | 4.60 e-03 | 0.99 | 0.01 | 0.01 | 0.95 |

**S4 Table: CONSORT Diagram**


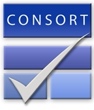
CONSORT 2010 checklist of information to include when reporting a randomised trial*

| Section/Topic | Item No | Checklist item | Reported on page No |  |  |
| --- | --- | --- | --- | --- | --- |
| Title and abstract | | | | | |
|  | 1a | Identification as a randomised trial in the title | 1 |  |  |
|  | 1b | Structured summary of trial design, methods, results, and conclusions (for specific guidance see CONSORT for abstracts) | 4 |  |  |
| Introduction | | | | | |
| Background and objectives | 2a | Scientific background and explanation of rationale | 6 |  |  |
|  | 2b | Specific objectives or hypotheses | 7 |  |  |
| Methods | | | | | |
| Trial design | 3a | Description of trial design (such as parallel, factorial) including allocation ratio | 8-9 & supp |  |  |
|  | 3b | Important changes to methods after trial commencement (such as eligibility criteria), with reasons | - |  |  |
| Participants | 4a | Eligibility criteria for participants | 8 & supp |  |  |
|  | 4b | Settings and locations where the data were collected | 9 |  |  |
| Interventions | 5 | The interventions for each group with sufficient details to allow replication, including how and when they were actually administered | 8-12 & supp |  |  |
| Outcomes | 6a | Completely defined pre-specified primary and secondary outcome measures, including how and when they were assessed | 7 |  |  |
|  | 6b | Any changes to trial outcomes after the trial commenced, with reasons | - |  |  |
| Sample size | 7a | How sample size was determined | 9 |  |  |
|  | 7b | When applicable, explanation of any interim analyses and stopping guidelines | - |  |  |
| Randomisation: |  |  |  |  |  |
| Sequence generation | 8a | Method used to generate the random allocation sequence | 10 & supp |  |  |
|  | 8b | Type of randomisation; details of any restriction (such as blocking and block size) | 10 & supp |  |  |
| Allocation concealment mechanism | 9 | Mechanism used to implement the random allocation sequence (such as sequentially numbered containers), describing any steps taken to conceal the sequence until interventions were assigned | 10 & supp |  |  |
| Implementation | 10 | Who generated the random allocation sequence, who enrolled participants, and who assigned participants to interventions | 9 & supp |  |  |
| Blinding | 11a | If done, who was blinded after assignment to interventions (for example, participants, care providers, those assessing outcomes) and how | 9 & supp |  |  |
|  | 11b | If relevant, description of the similarity of interventions | - |  |  |
| Statistical methods | 12a | Statistical methods used to compare groups for primary and secondary outcomes | 11-12 |  |  |
|  | 12b | Methods for additional analyses, such as subgroup analyses and adjusted analyses | 11-12 |  |  |
| Results | | | | | |
| Participant flow (a diagram is strongly recommended) | 13a | For each group, the numbers of participants who were randomly assigned, received intended treatment, and were analysed for the primary outcome | 15 & Sup Fig2 |  |  |
|  | 13b | For each group, losses and exclusions after randomisation, together with reasons | 15 & Sup Fig2 |  |  |
| Recruitment | 14a | Dates defining the periods of recruitment and follow-up | 10 & Sup Fig2 |  |  |
|  | 14b | Why the trial ended or was stopped | - |  |  |
| Baseline data | 15 | A table showing baseline demographic and clinical characteristics for each group | Table 1 |  |  |
| Numbers analysed | 16 | For each group, number of participants (denominator) included in each analysis and whether the analysis was by original assigned groups | Sup Fig2 |  |  |
| Outcomes and estimation | 17a | For each primary and secondary outcome, results for each group, and the estimated effect size and its precision (such as 95% confidence interval) | 13 |  |  |
|  | 17b | For binary outcomes, presentation of both absolute and relative effect sizes is recommended | - |  |  |
| Ancillary analyses | 18 | Results of any other analyses performed, including subgroup analyses and adjusted analyses, distinguishing pre-specified from exploratory | - |  |  |
| Harms | 19 | All important harms or unintended effects in each group (for specific guidance see CONSORT for harms) | 10 |  |  |
| Discussion | | | | | |
| Limitations | 20 | Trial limitations, addressing sources of potential bias, imprecision, and, if relevant, multiplicity of analyses | 15 |  |  |
| Generalisability | 21 | Generalisability (external validity, applicability) of the trial findings | 16 |  |  |
| Interpretation | 22 | Interpretation consistent with results, balancing benefits and harms, and considering other relevant evidence | 16-17 |  |  |
| Other information | | | | |  |
| Registration | 23 | Registration number and name of trial registry | 8 |  |  |
| Protocol | 24 | Where the full trial protocol can be accessed, if available | 8 |  |  |
| Funding | 25 | Sources of funding and other support (such as supply of drugs), role of funders | 3 |  |  |

1. Rosenfield, D., et al., *Changes in Dosing and Dose Timing of D-Cycloserine Explain Its Apparent Declining Efficacy for Augmenting Exposure Therapy for Anxiety-related Disorders: An Individual Participant-data Meta-analysis.* J Anxiety Disord, 2019. **68**: p. 102149.

2. Onur, O.A., et al., *The N-Methyl-D-Aspartate Receptor Co-agonist D-Cycloserine Facilitates Declarative Learning and Hippocampal Activity in Humans.* Biological Psychiatry, 2010. **67**(12): p. 1205-1211.

3. Reinecke, A., et al., *Neurocognitive processes in d-cycloserine augmented single-session exposure therapy for anxiety: A randomized placebo-controlled trial.* Behaviour Research and Therapy, 2020. **129**: p. 103607.

4. Ressler, K.J., et al., *Cognitive enhancers as adjuncts to psychotherapy: use of D-cycloserine in phobic individuals to facilitate extinction of fear.* Arch Gen Psychiatry, 2004. **61**(11): p. 1136-44.

5. Rodebaugh, T.L., C.A. Levinson, and E.J. Lenze, *A high-throughput clinical assay for testing drug facilitation of exposure therapy.* Depression and anxiety, 2013. **30**(7): p. 631-637.

6. Guastella, A.J., et al., *A randomized controlled trial of D-cycloserine enhancement of exposure therapy for social anxiety disorder.* Biol Psychiatry, 2008. **63**(6): p. 544-9.

7. Hofmann, S.G., et al., *Augmentation of exposure therapy with D-cycloserine for social anxiety disorder.* Arch Gen Psychiatry, 2006. **63**(3): p. 298-304.

8. Nave, A.M., D.F. Tolin, and M.C. Stevens, *Exposure therapy, D-cycloserine, and functional magnetic resonance imaging in patients with snake phobia: a randomized pilot study.* J Clin Psychiatry, 2012. **73**(9): p. 1179-86.

9. Herigstad, M., et al., *Treating breathlessness via the brain: changes in brain activity over a course of pulmonary rehabilitation.* Eur Respir J, 2017. **50**(3).

10. Finnegan, S.L., et al., *Brain activity measured by functional brain imaging predicts breathlessness improvement during pulmonary rehabilitation.* Thorax, 2023. **78**(9): p. 852.

11. Levy, M.L., et al., *Diagnostic spirometry in primary care: Proposed standards for general practice compliant with American Thoracic Society and European Respiratory Society recommendations: a General Practice Airways Group (GPIAG)1 document, in association with the Association for Respiratory Technology & Physiology (ARTP)2 and Education for Health3 1* [*www.gpiag.org*](file:///Users/sarahfinnegan/Desktop/Postdoc/Manuscripts/StructuralPaper/PlosOne/www.gpiag.org) *2* [*www.artp.org*](file:///Users/sarahfinnegan/Desktop/Postdoc/Manuscripts/StructuralPaper/PlosOne/www.artp.org) *3* [*www.educationforhealth.org.uk*](file:///Users/sarahfinnegan/Desktop/Postdoc/Manuscripts/StructuralPaper/PlosOne/www.educationforhealth.org.uk)*.* Prim Care Respir J, 2009. **18**(3): p. 130-47.

12. Bradley, J., et al., *Validity of a modified shuttle test in adult cystic fibrosis.* Thorax, 1999. **54**(5): p. 437-9.

13. Mahler, D.A., et al., *Comparison of clinical dyspnea ratings and psychophysical measurements of respiratory sensation in obstructive airway disease.* Am Rev Respir Dis, 1987. **135**(6): p. 1229-33.

14. Douaud, G., et al., *Anatomically related grey and white matter abnormalities in adolescent-onset schizophrenia.* Brain, 2007. **130**(Pt 9): p. 2375-86.

15. Smith, S.M., et al., *Advances in functional and structural MR image analysis and implementation as FSL.* Neuroimage, 2004. **23 Suppl 1**: p. S208-19.

16. Andersson J.L.R, J.M., Smith S., *Non-linear optimisation.* FMRIB technical report, 2007.
